# Quorum sensing regulation by the nitrogen phosphotransferase system in *Pseudomonas aeruginosa*

**DOI:** 10.1128/jb.00048-25

**Published:** 2025-08-01

**Authors:** Samalee Banerjee, Nicole E. Smalley, Pradtahna Saenjamsai, Anthony R. Fehr, Ajai A. Dandekar, Matthew T. Cabeen, Josephine R. Chandler

**Affiliations:** 1 Department of Molecular Biosciences, University of Kansas, Lawrence, Kansas, USA; 2 Department of Microbiology, University of Washington 312771, Seattle, Washington, USA; 3 Department of Microbiology and Molecular Genetics, Oklahoma State University 539937 , Stillwater, Oklahoma, USA; Geisel School of Medicine at Dartmouth, Hanover, New Hampshire, USA

**Keywords:** phosphotransferase, *Pseudomonas aeruginosa*, quorum sensing, virulence regulation

## Abstract

In the opportunistic pathogen *Pseudomonas aeruginosa*, the nitrogen-related phosphotransferase system (PTS^Ntr^) influences multiple virulence behaviors. The PTS^Ntr^ is comprised of three enzymes: first PtsP, then the PtsO phosphocarrier, and the final PtsN phosphoacceptor. We previously showed that *ptsP* inactivation enhances LasI-LasR quorum sensing, a system by which *P. aeruginosa* regulates genes in response to population density. LasI synthesizes a diffusible autoinducer that binds and activates the LasR receptor, which activates a feedback loop by increasing *lasI* expression. In this study, we examined the impact of the PTS^Ntr^ on quorum sensing. Disruption of *ptsP* increased the expression of some, but not all, tested quorum-controlled genes, including *lasI, phzM* (pyocyanin biosynthesis), *hcnA* (hydrogen cyanide biosynthesis), and, to a lesser extent, *rsaL* (quorum sensing regulator). Expression of these genes remained dependent on LasR and the autoinducer, whether provided endogenously or exogenously. Increased *lasI* expression in ∆*ptsP* (or ∆*ptsO*) cells was partly due to the presence of unphosphorylated PtsN, which alone was sufficient to elevate *lasI* expression. However, we observed residual increases in ∆*ptsP* or ∆*ptsO* cells even in the absence of PtsN, suggesting that PtsP and PtsO can regulate gene expression independently of PtsN. Indeed, genetically disrupting the PtsO phosphorylation site impacted gene expression in the absence of PtsN, and transcriptomic evidence suggested that PtsO and PtsN have distinct regulons. Our results expand our view of how the PTS^Ntr^ components function both within and apart from the classic phosphorylation cascade to regulate key virulence behaviors in *P. aeruginosa*.

**IMPORTANCE:**

*Pseudomonas aeruginosa* often causes severe and difficult-to-treat infections. *P. aeruginosa* virulence requires the nitrogen-related phosphotransferase system (PTS^Ntr^), which comprises the phosphocarrier proteins PtsP and PtsO and the final phosphoacceptor, PtsN. The PTS^Ntr^ is known to modulate quorum sensing, but little is known about the mechanism of regulation. Here, we examined quorum sensing regulation by the PTS^Ntr^. We showed that the PTS^Ntr^ increases quorum sensing-mediated activation of certain genes through the additive effects of both PtsO and PtsN. We also used transcriptomics to determine the regulons of PtsO and PtsN and found that they are largely nonoverlapping. The results position PtsO and PtsN as independent effectors in the PTS^Ntr^ and shed new light on virulence regulation in this important pathogen.

## INTRODUCTION

*Pseudomonas aeruginosa* is a gram-negative, opportunistic pathogen found in many habitats, particularly those linked to human activity (1). *P. aeruginosa* causes severe and sometimes fatal infections in patients with cystic fibrosis, acute leukemia, burn wounds, and organ transplants. It is also commonly contracted in healthcare settings (2). There is a significant global health burden from *P. aeruginosa* infections, which are thought to be responsible for over $700 million in health-related costs annually (3). *P. aeruginosa* infections are particularly difficult to treat due to the prevalence of multidrug-resistant strains, an arsenal of virulence factors, and its ability to adapt and survive in diverse environments (4–6).

In *P. aeruginosa,* several key virulence factors are regulated by the nitrogen-related phosphotransferase system (PTS^Ntr^) (7). This system was first described in *Escherichia coli* as important for regulating changes in metabolism in response to the available ratio of carbon and nitrogen (8). The PTS^Ntr^ regulates diverse behaviors in different bacteria; for example, in *E. coli*, the PTS^Ntr^ regulates metabolism (9) and potassium transport (10), and the *Pseudomonas putida* PTS^Ntr^ regulates toluene degradation (11) and polyhydroxyalkanoates (12). The PTS^Ntr^ is paralogous to the canonical sugar PTSs that phosphorylate and import saccharides. The first enzyme of the PTS^Ntr^ is PtsP (“enzyme I” or EI, which is analogous to the sugar-PTS EI enzymes). PtsP transfers phosphate, thought to be taken from phosphoenolpyruvate (PEP), to the second enzyme, PtsO (“NPr,” analogous to the sugar PTS histidine protein HPr). PtsO then transfers phosphate to the final enzyme PtsN (“enzyme IIA” or EIIA, analogous to EIIA of the sugar PTS). A hallmark of the PTS^Ntr^ system, when compared with sugar PTSs, is that the EI has a GAF domain (named after some of the proteins it is found in; c**G**MP-specific phosphodiesterases, **a**denylyl cyclases, and **F**hlA) (13); GAF domains directly bind small molecule ligands and subsequently affect a response (14). In *P. aeruginosa* and other bacteria, the *ptsO* and *ptsN* genes are located downstream of the nitrogen-related sigma factor gene *rpoN,* whereas the *ptsP* gene is located at a distant site (15).

 The *P. aeruginosa* PTS^Ntr^ is important for virulence, although its virulence effects are not well understood. Deleting the first gene, *ptsP,* attenuates *P. aeruginosa* pathogenicity in infections of mice, *Caenorhabditis elegans,* and plant leaves (16, 17). In mice, *ptsP* null mutations decrease resistance to host innate immunity (18). In laboratory evolution experiments, *ptsP* mutations increase resistance to the clinically important antibiotic tobramycin (19–21) through an unknown mechanism. In addition, the PTS^Ntr^ system impacts *P. aeruginosa* biofilm formation by modulating the Pel polysaccharide (22). Studies of PtsN also suggest that PtsO acts as a “specificity factor” to ensure that PtsN is not phosphorylated by another PTS EI, FruB (from the fructose PTS system), and that modulation of PtsN phosphorylation impacts the expression of dozens of genes, including many that are virulence-associated (7).

*ptsP* disruption also activates transcription of the quorum sensing signal synthase gene *lasI* (21, 23). Quorum sensing is a population density-dependent communication system (for reviews, see refs [24, 25]). In *P. aeruginosa,* LasI synthesizes the acyl-homoserine lactone (AHL) signal molecule *N-*(3-oxo)-dodecanoyl L-homoserine lactone (3OC12-HSL), which is detected by the signal receptor LasR. Upon binding, LasR activates the transcription of dozens of genes, including *lasI,* which creates a positive feedback loop. In addition to the LasR-LasI system, there is a second AHL signal-receptor pair in *P. aeruginosa,* RhlR-RhlI, for which the signal is *N-*butanoyl L-homoserine lactone (C4-HSL). Together, these systems activate the production of virulence factors such as pyocyanin, protease, rhamnolipids, hydrogen cyanide, biofilm matrix proteins, lectin, and alkaline protease, and they have been shown to be important for virulence in numerous infection models (16, 26–29).

Although a regulatory link between quorum sensing and the PTS^Ntr^ has been established (21, 23), the mechanism of this regulation is not well understood. Using transcriptional reporters, we determined that *ptsP* disruption influences only a subset of quorum sensing-regulated genes. By adding exogenous 3OC12-HSL using an inducible *lasI* strain, we showed that *ptsP* disruption increases LasR-dependent gene activation in response to both endogenous and exogenous 3OC12-HSL, suggesting that the ∆*ptsP*-dependent increase in endogenous 3OC12-HSL is not responsible for the effects of this mutation on gene regulation. We also demonstrated independent, but additive, effects of PtsO and PtsN on *lasI* expression; the conserved phosphorylation sites of each protein were important for differential regulation. Transcriptomics studies of strains in which the PtsO enzyme phosphorylation state varied uncovered a PtsO-dependent, distinct regulon that did not overlap with that of PtsN. The results implicate PtsO and PtsN as independent outputs of the PTS^Ntr^, highlighting the complexity of this important virulence determinant in *P. aeruginosa*.

## RESULTS

### *ptsP* deletion increases the expression of some, but not all, LasR-controlled genes

**Fig 1:**
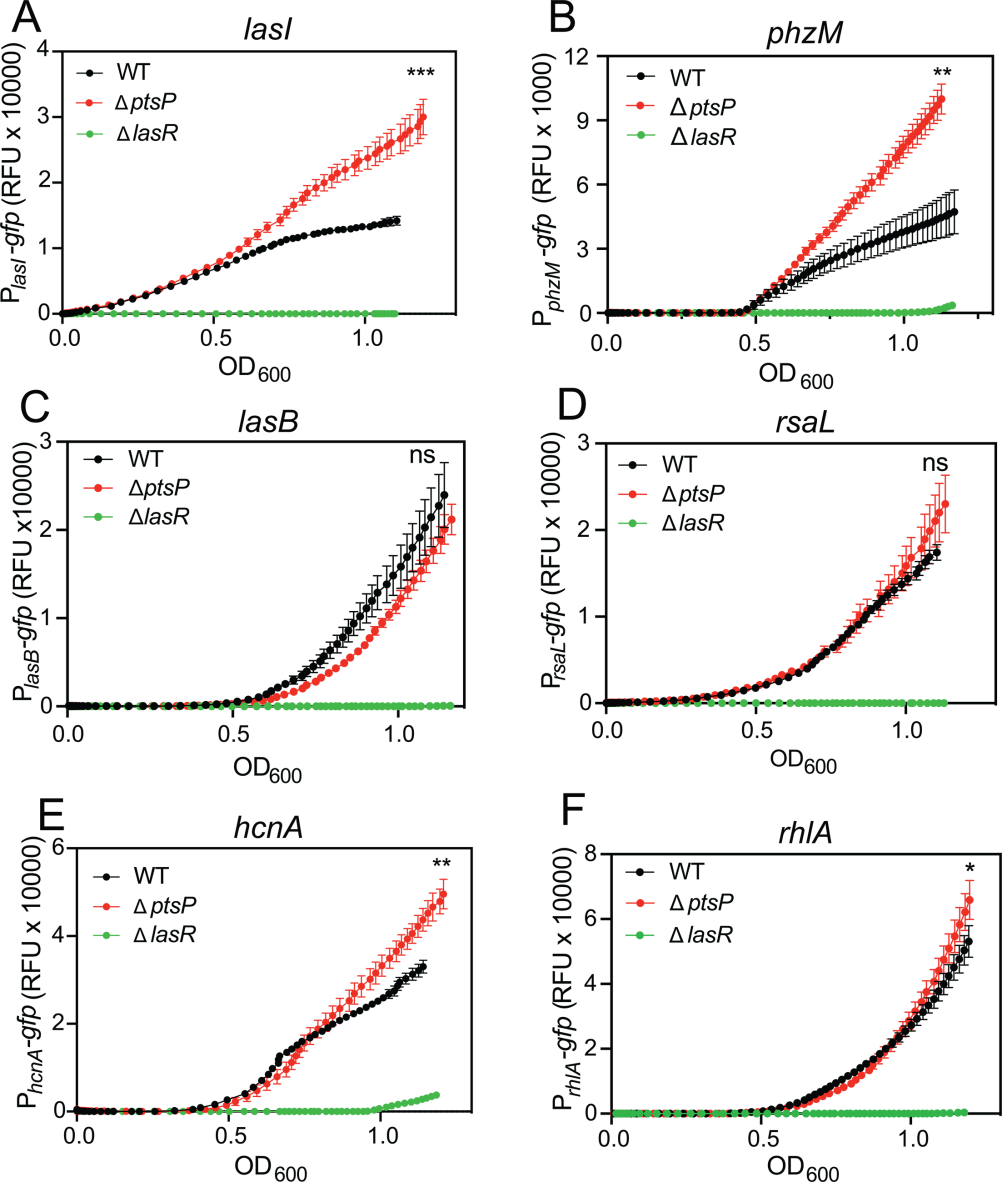
∆*ptsP* influence on quorum sensing gene transcription. GFP fluorescence was measured in strains harboring reporters to the *lasI* (**A**), *phzM* (**B**), *lasB* (**C**), *rsaL* (**D**), *hcnA* (**E**), and *rhlA* (**F**) promoters in PA14, PA14 *∆ptsP,* and PA14 ∆*lasR*. Fluorescence output was measured over a time course using a BioTek plate reader. Data points are the means of three replicates, and the error bars represent the standard deviation. Significance by *t*-test of wild type compared with ∆*ptsP* from the final time point (OD_600_-adjusted fluorescence values); ***, *P* < 0.0005; **, *P* < 0.005; *, *P* < 0.05; ns, not significant.

To better understand the role of *ptsP-*null mutations on LasR activity, we used a pP*_lasI_-gfp* reporter plasmid (30), which contains the promoter of the LasR-regulated *lasI* gene fused to a gene encoding GFP. We transformed the pP*_lasI_-gfp* plasmid into *P. aeruginosa* ∆*ptsP* and ∆*lasR* and compared fluorescence intensities over time (Fig. 1A). These deletion mutants grew identically to the wild-type strain, PA14 (Fig. S1). Consistent with our prior studies, at the end of the experiment (14 h), we observed ~3-fold higher P*_lasI_-gfp* activation in ∆*ptsP* compared with wild type (Fig. 1A, unpaired *t*-test, *P* < 0.001); there was no activation in the absence of LasR. This difference was observed after cultures reached an OD_600_ of ~0.6, which correlates with the initiation of the stationary phase (Fig. S1). We asked if higher *lasI* expression levels were due to the ∆*ptsP* mutation using two complementary approaches. First, we quantified *lasI* transcripts from stationary-phase cells without the reporter present and found that *lasI* transcripts were 3-fold higher in ∆*ptsP* cultures compared with wild type (*P* < 0.02, Fig. S2A). We also measured 3OC12-HSL concentrations in wild-type and ∆*ptsP* cultures after 18 h growth. We found that 3OC12-HSL levels were almost 5-fold higher for the ∆*ptsP* strain compared with that of the wild type (Fig. S2B). These results are both consistent with the observed difference in *lasI-gfp* reporter activation due to the ∆*ptsP* mutation.

We sought to test if *ptsP* disruption similarly regulated the expression of other quorum sensing-regulated genes. We selected promoters for the following genes: *phzM*, encoding a key enzyme in phenazine biosynthesis, based on prior studies showing the PTS^Ntr^ regulation of phenazine production (21, 23); *lasB* (elastase, a protease), *rsaL* (repressor of LasR-I system), *hcnA* (hydrogen cyanide biosynthesis), and *rhlA* (rhamnolipid surfactant) (31). The latter genes were selected because they represent a suite of genes regulated by LasR and RhlR (32). In addition, LasR directly binds the promoters of *lasB, rsaL,* and *hcnA* (32). We engineered plasmid-based reporters of each of these gene promoters (pP*_phzM_-gfp,* pP*_lasB_-gfp,* pP*_rsaL_-gfp,* pP*_hcnA_-gfp,* and pP*_rhlA_-gfp*). As expected, the expression of each of these genes was dependent on LasR in our time course experiments (Fig. 1). Compared with the wild type, in the ∆*ptsP* strain, we observed higher activation of the *phzM, hcnA,* and, to a lesser degree, *rhlA* reporters. These results show that disrupting *ptsP* increases the expression of some, but not all, quorum sensing-regulated genes.

### The ∆*ptsP-*dependent increase in 3OC12-HSL is not responsible for changes in gene transcription

**Fig 2:**
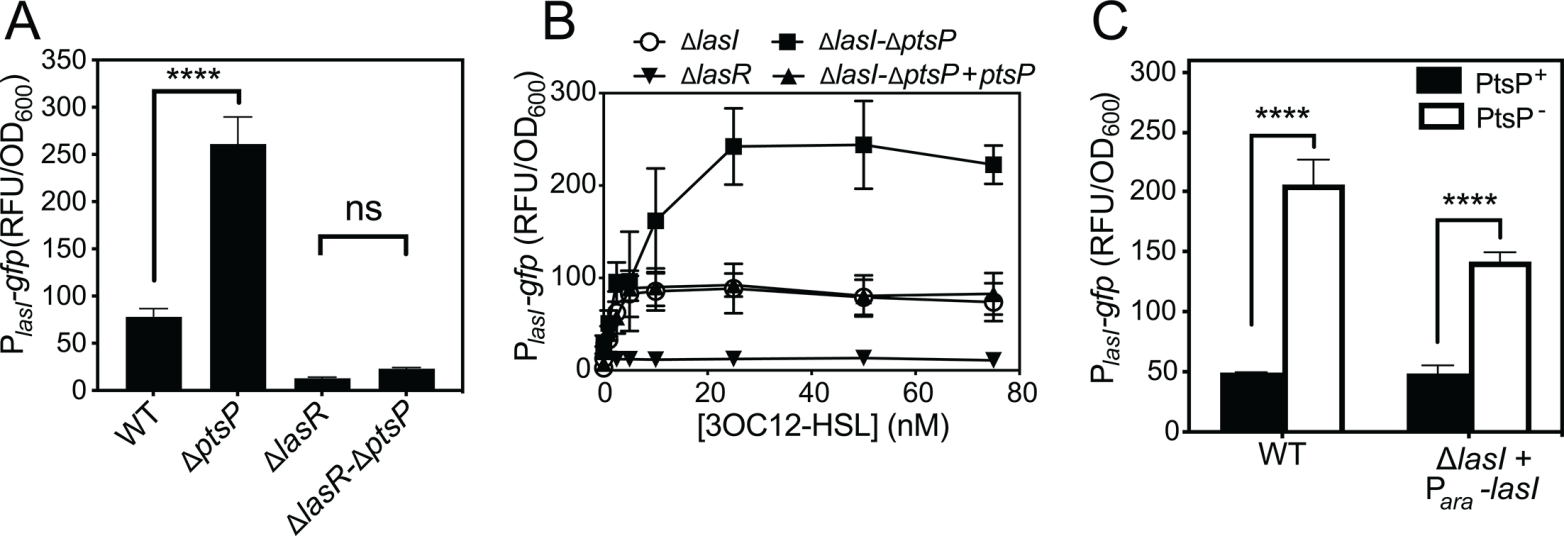
Role of LasR and exogenous 3OC12-HSL on ∆*ptsP-*dependent *lasI* activation. (A) Growth-adjusted GFP fluorescence in strains harboring the P*_lasI_-gfp* reporter plasmid. Fluorescence was measured after 18 h growth in test-tube grown cultures. The results are of wild-type (WT, strain PA14) or isogenic mutants as indicated. For panels B and C, the strains carried a chromosomally inserted CTX-1 cassette (CTX), CTX plus *ptsP,* or CTX plus an arabinose-inducible *lasI* (P_*ara*_-*lasI*). For (**B**), strains were grown in different concentrations of 3OC12-HSL, and fluorescence was measured. For (**C**), black bars indicate strains with the native *ptsP* gene intact, and white bars indicate strains with ∆*ptsP*; 0.2% arabinose was added to all cultures. Data points are the means of three replicates, and the error bars represent the standard deviation. Statistical significance was determined by *t*-tests; ***, *P* < 0.0005; ns, not significant.

We sought to gain further insight into the relationship between PtsP and *lasI* regulation. We first asked if LasR is required for ∆*ptsP-*dependent activation of *lasI* transcription. We deleted *lasR* from a ∆*ptsP* mutant and measured *lasI* expression levels using our pP*_lasI_-gfp* reporter. In the absence of *lasR,* we observed no significant difference in *lasI* activation in a ∆*ptsP* mutant compared with wild type (Fig. 2A), supporting the idea that elevated *lasI* expression in a ∆*ptsP* mutant requires LasR.

LasR responds to 3OC12-HSL, and the ∆*ptsP* mutation increases 3OC12-HSL levels (Fig. S2B). Thus, we next asked if increased 3OC12-HSL levels are responsible for ∆*ptsP-*dependent effects on gene transcription. We used two approaches to address this possibility. First, we deleted *lasI* from our wild-type and ∆*ptsP* mutant strains and grew these strains with varying concentrations of exogenously added 3OC12-HSL (Fig. 2B). With the pP*_lasI_-gfp* reporter, the minimum 3OC12-HSL concentration with which we could detect a response was 1 nM. The response was saturated at concentrations of 25 nM and higher. In this concentration range, we observed a ~3-fold increase in GFP levels in the ∆*ptsP* strain at all concentrations. We could restore GFP levels to that of the wild type in this strain by inserting an intact copy of the *ptsP* gene into the neutral *att* site in the chromosome. We also engineered strains to constitutively express *lasI* using an arabinose-inducible promoter (P*_araBAD_-gfp*), which we inserted into the neutral *att* site in the chromosome of the ∆*lasI* and ∆*lasI-*∆*ptsP* mutants. This approach unlinks *lasI* expression from LasR control (33). With these strains, we also observed a ~ 3-fold increase in P*_lasI_-gfp* activation due to the ∆*ptsP* mutation (Fig. 2C). The ∆*lasI* and ∆*lasI-*∆*ptsP* strains each harboring P*_ara_-lasI* produced 3OC12-HSL at concentrations of 254 nM and 198 nM, respectively; this concentration was sufficient to maximally induce the GFP reporter (Fig. 2B). From these results, we conclude that increased production of 3OC12-HSL is not responsible for the effects on transcription in the ∆*ptsP* mutant.

### Deleting the PtsP GAF domain does not impact *lasI* expression

**Fig 3:**
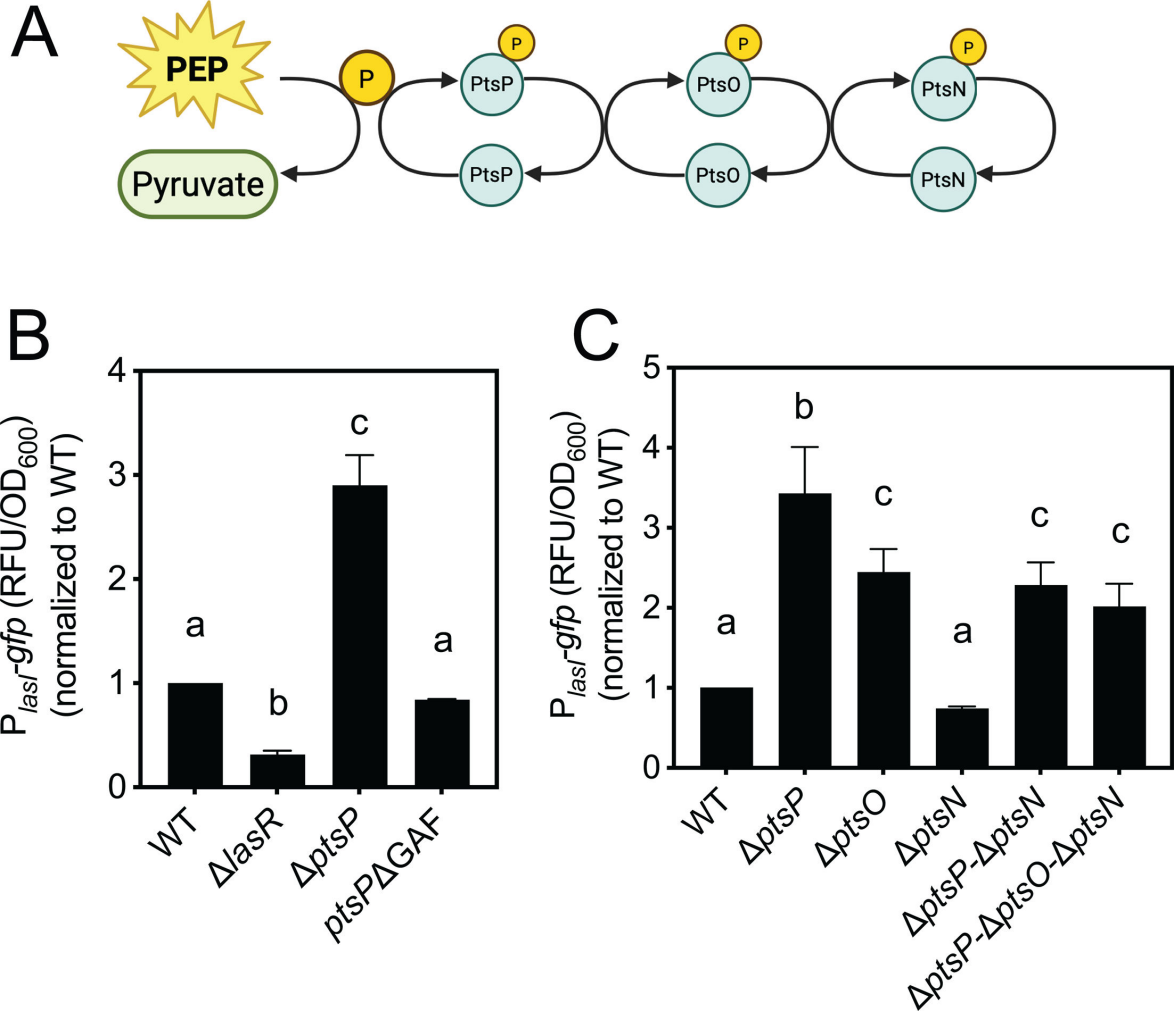
The PtsO and PtsN enzymes, but not the PtsP GAF domain, contribute to *lasI* regulation. (**A**) Illustration of the PTS^Ntr^ phosphorylation cascade. Phosphate is transferred by PtsP (EI Ntr) from phosphoenolpyruvate (PEP) to PtsO (Npr) and in turn to PtsN (EII Ntr), which has unknown regulatory targets. The ∆*ptsP* mutation is expected to result in constitutively unphosphorylated states of PtsO and PtsN. (**B**) and (**C**) Transcription from the *lasI* promoter was monitored as GFP fluorescence in cells transformed with the pP*_lasI_-gfp* reporter plasmid. WT, wild-type strain PA14. *ptsP*∆GAF is an allelic replacement of *ptsP* with a deletion of the sequence encoding the GAF domain. Data shown are growth-adjusted fluorescence (**B**) or growth-adjusted fluorescence normalized to wild type (**C**) after 18 h growth. The results show the means of three independent experiments, and vertical bars represent the standard deviation. Statistical significance was determined using a one-way ANOVA with Tukey’s multiple comparisons *post-hoc* test; different letters indicate *P* < 0.05, and the same letters indicate *P* > 0.05.

In *E. coli,* the phosphorylation state of PtsP appears to depend on the cellular ratio of nitrogen to carbon, which is detected by direct binding of glutamine and α-ketoglutarate by PtsP through the GAF domain (Fig. 3A). In *P. aeruginosa,* the PtsP GAF domain is important for PtsN phosphorylation (7) but does not impact biofilm formation (22). We thus asked whether the PtsP GAF domain is required for its role in *lasI* regulation. We constructed a PA14 strain in which we deleted the GAF domain from *ptsP* at its native site in the genome (*ptsP*∆GAF). We introduced our pP*_lasI_-gfp* reporter plasmid to this *ptsP*∆GAF strain and compared *lasI* transcription activation with that of the wild-type and a full *ptsP* deletion mutant. As expected, *lasI* expression increased in the ∆*ptsP* strain; however, deleting only the GAF domain did not similarly increase *lasI* expression (Fig. 3B). We also tested whether the PtsP GAF domain is required for PtsP’s role in regulating the *phzM* or *hcnA* promoters by using the pP*_phzM_-gfp* and pP*_hcnA_-gfp* reporter plasmids, respectively. As with *lasI,* we did not observe a significant role for the PtsP GAF domain in regulating *phzM* or *hcnA* expression (Fig. S3). These results showed that the GAF domain is dispensable for the role of PtsP in regulating *lasI, phzM,* and *hcnA.*

### *lasI* is regulated by other enzymes in the PTS^Ntr^ pathway

Next, we examined the role of PtsO and PtsN, the other two enzymes in the PTS^Ntr^ phosphotransfer system (Fig. 3A), in *lasI* regulation. As a first step, we introduced the P*_lasI_-gfp* reporter plasmid to wild-type PA14 and single, double, and triple deletion mutant(s) of the three PTS^Ntr^ genes *ptsP*, *ptsO,* and *ptsN*. The results are in Fig. 3C and summarized in Table S1. Deleting *ptsO* from the wild-type genome increased *lasI* expression but not as much as that of the ∆*ptsP* mutant (~2-fold for ∆*ptsO* and ~3-fold for ∆*ptsP*). In the ∆*ptsO* mutant, we could restore *lasI* expression to that of the wild type by introducing a functional copy of *ptsO* into the genome (Fig. S4). These results showed that *lasI* is regulated, in part, by PtsO. Deleting *ptsN* from the wild-type genome did not significantly alter *lasI* expression (Fig. 3C); however, deleting *ptsN* from the ∆*ptsP* mutant decreased *lasI* expression but not to wild-type levels. Thus, PtsN regulates transcription from the *lasI* promoter, but only in the absence of PtsP. In a strain in which all three of the PTS^Ntr^ enzymes are deleted (∆*ptsP-*∆*ptsO-*∆*ptsN*), we observed an increase in *lasI* expression compared with the wild type. Together, the results support the idea that PTS^Ntr^ may have regulatory effects on *lasI* expression that are both activating (in the absence of PtsP or PtsO) and suppressing (when PtsN, or PtsO and PtsN, are absent in a Δ*ptsP* background).

### Unphosphorylated PtsN activates *lasI* expression

**Fig 4:**
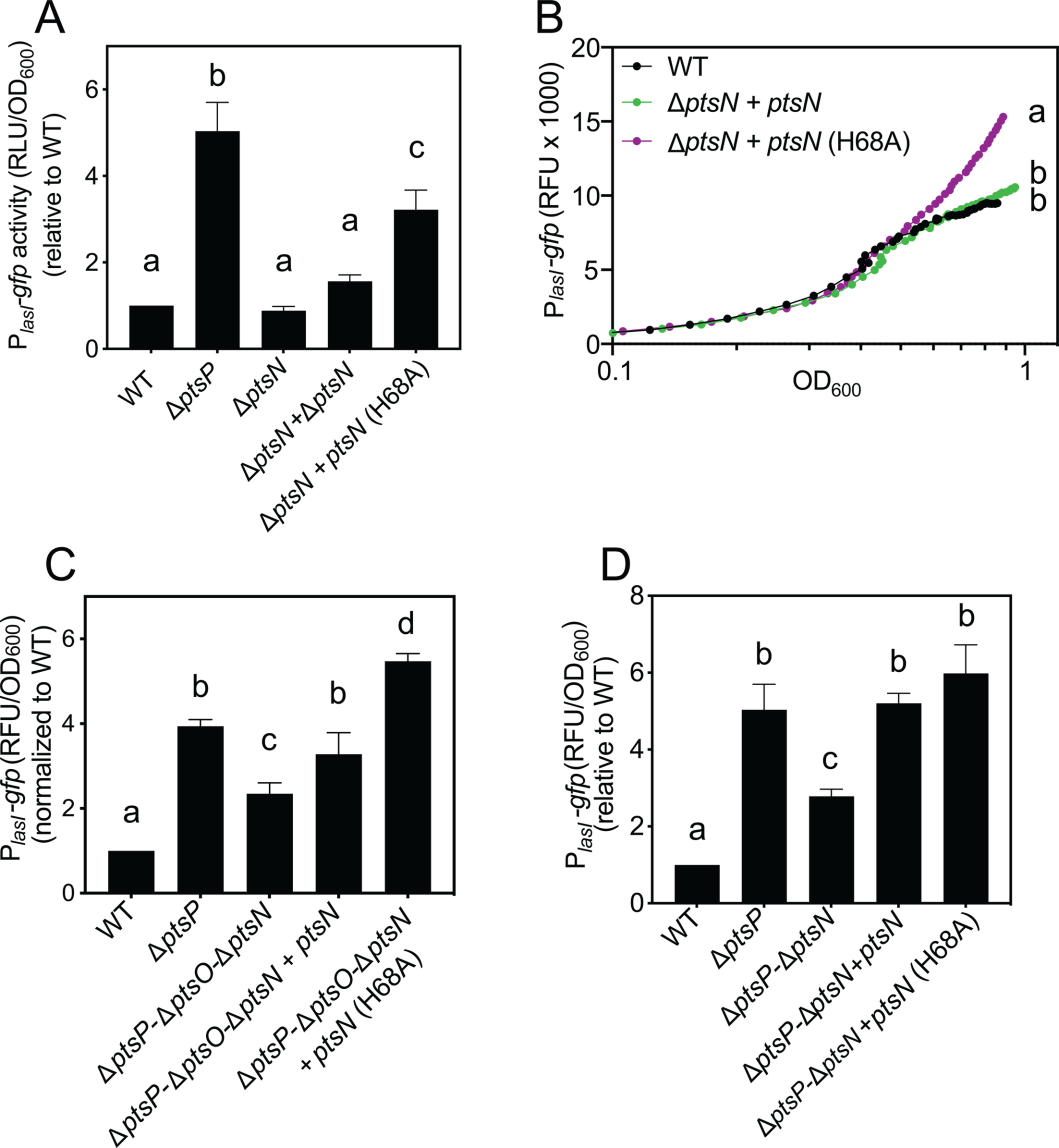
Mutation of the PtsN phosphorylation site increases *lasI* expression. Transcription from the *lasI* promoter was monitored as GFP fluorescence in cells transformed with the pP*_lasI_-gfp* reporter plasmid. Panels A, C, and D show growth-adjusted fluorescence normalized to wild type after 18 h of growth, and panel B shows fluorescence output measured over a time course using a BioTek plate reader. Strains carried a chromosomally inserted CTX-1 cassette (CTX), CTX plus the wild type *ptsN,* or CTX plus the H68A variant of PtsN (*ptsN* [H68A]). Data are means of three replicates, and vertical bars represent standard deviation. For panel B, the standard deviation was <10% of the means for all strains and time points, which was too small to be seen on the graph. Statistical significance was determined using a one-way ANOVA with Tukey’s multiple comparisons *post-hoc* test; different letters indicate *P* < 0.05, and the same letters indicate *P* > 0.05. For panel B, significance was of growth-adjusted fluorescence values from the final time point.

PtsN is unphosphorylated in the absence of PtsP (7), leading us to posit that *lasI* activation in a ∆*ptsP* mutant may be at least partially due to unphosphorylated PtsN. This model is consistent with our finding that deleting *ptsN* from a ∆*ptsP* mutant reduces *lasI* expression in this strain (Fig. 3C). To test this hypothesis, we utilized a PtsN allele that harbors a single amino acid substitution (H68A) that changes its phosphorylation site to alanine. This substitution has been shown to effectively block PtsN phosphorylation in *P. aeruginosa* without affecting its cellular abundance (7). We moved the unmutated PtsN or the PtsN^H68A^ genes in single copy to the chromosome of the ∆*ptsN* mutant and used the pP*_lasI_-gfp* reporter plasmid to compare *lasI* expression in these strains with that of the ∆*ptsN* and wild-type strains (Fig. 4; Table S1). Consistent with our earlier result, *lasI* expression was similar in the wild-type and ∆*ptsN* mutants. In the ∆*ptsN* strain, we found that PtsN^H68A^, but not the wild-type PtsN, increased *lasI* expression by ~3-fold (Fig. 4A and B); this difference was observed after cultures reached the stationary phase, with no significant effects on growth (OD_600_ ~0.6; Fig. S5). We also assessed the role of PtsN and PtsN^H68A^ in regulating *lasI* expression in the ∆*ptsP-*∆*ptsO-*∆*ptsN* mutant, where we could evaluate regulation effects in the absence of the other PTS^Ntr^ enzymes. In this genetic background, we expected *lasI* expression to be activated by ectopically expressing PtsN^H68A^, as observed when we expressed this allele in the ∆*ptsN* strain. We also expected *lasI* expression to be similarly activated with wild-type PtsN in this strain because PtsN is assumed to be unphosphorylated in the absence of *ptsP*. Consistent with our expectations, PtsN^H68A^ increased *lasI* expression levels ~ 2.4-fold compared with no PtsN alleles. However, wild-type PtsN increased levels to a lesser degree than that of the PtsN^H68A^ allele, by only ~1.4-fold (Fig. 4C). We posited that PtsN may be partially phosphorylated in this strain by FruB, the fructose EI enzyme, which can phosphorylate PtsN in the absence of PtsO (7). Thus, we also examined the role of PtsN and PtsN^H68A^ on *lasI* expression in a ∆*ptsP-*∆*ptsN* double mutant, where PtsO is intact. In this strain, transcription from the *lasI* promoter was increased to the same level by ectopically expressing either PtsN^H68A^ or PtsN (Fig. 4D). Together, these results support the conclusion that unphosphorylated PtsN activates *lasI* expression, and disrupting *ptsP* results in elevated transcription levels due to an accumulation of unphosphorylated PtsN.

### In the absence of PtsN, *lasI* expression is repressed by both PtsP and PtsO

**Fig 5:**
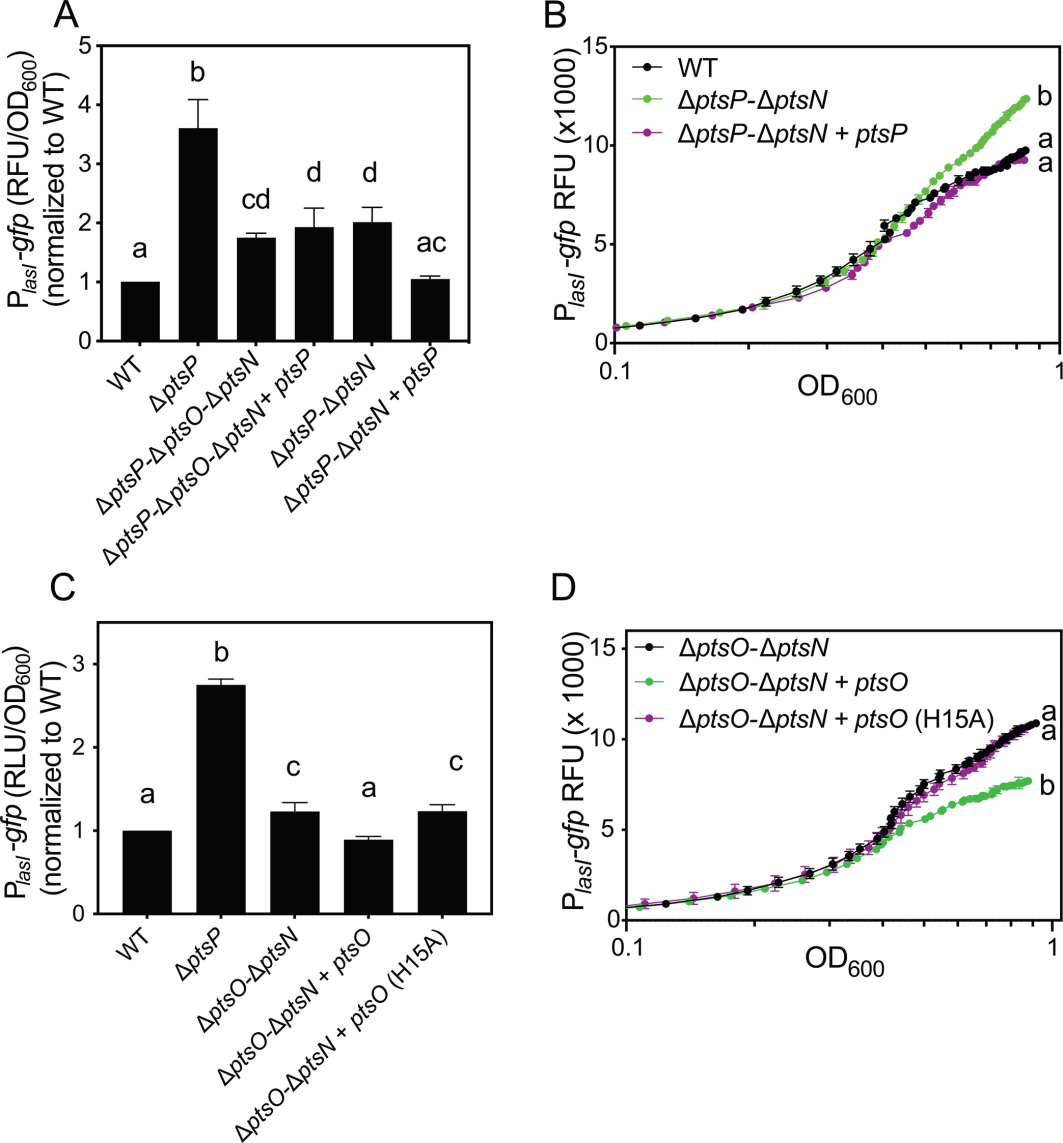
*lasI* regulation by PtsP and PtsO. Transcription from the *lasI* promoter was monitored as GFP fluorescence in cells transformed with the pP*_lasI_-gfp* reporter plasmid. (**A and C**) Growth-adjusted fluorescence normalized to wild type after 18 h of growth. (**B and D**) Fluorescence output was measured over a time course using a BioTek plate reader. Strains carried a chromosomally integrated CTX-1 cassette (CTX), CTX plus the wild-type *ptsO,* or CTX plus the H15A variant of PtsO (*ptsO* [H15A]). Data are means of three replicates, and vertical bars represent standard deviation. For panels B and D, the standard deviation was <10% of the means for all strains and time points, which was too small to be seen on the graph. Statistical significance was by one-way ANOVA with Tukey’s multiple comparisons *post-hoc* test; different letters indicate *P* < 0.05, and the same letters indicate *P* > 0.05. For panels B and D, the significance was of growth-adjusted fluorescence values from the final time point.

In our experiments, *lasI* reporter activity was significantly lower in the ∆*ptsN* mutant than in the ∆*ptsP-*∆*ptsN* mutant (Fig. 3C), suggesting that there is another mechanism of PTS^Ntr^ regulation that may be through PtsP or PtsO. Thus, we sought to unravel this additional regulation mechanism. We reasoned that if *lasI* is repressed by PtsP only, we should observe a PtsP-dependent reduction of *lasI* reporter activity in the absence of both PtsO and PtsN. To test this hypothesis, we moved *ptsP* in a single copy into the chromosome of the ∆*ptsP-*∆*ptsO-*∆*ptsN* triple mutant and transformed these strains with the P*_lasI_-gfp* plasmid reporter to measure transcription from the *lasI* promoter. We found that *ptsP* had no effect on *lasI* expression in this strain (Fig. 5A), showing that PtsP alone does not modulate *lasI* expression. However, in the ∆*ptsP-*∆*ptsN* mutant (where *ptsO* is intact), *ptsP* significantly decreased *lasI* expression (Fig. 5A and B); differences in expression, but not growth, were observed after the cultures reached stationary phase (OD_600_ ~0.6; Fig. 5B; Fig. S6A). These results suggest that PtsP can repress *lasI* expression but only in the presence of PtsO. To determine whether PtsO can regulate *lasI* in the absence of PtsP and PtsN, we compared *lasI* expression in the ∆*ptsP-*∆*ptsO-*∆*ptsN* triple mutant with that of the ∆*ptsP-*∆*ptsN* double mutant (where *ptsO* is intact); however, *lasI* expression in these two strains was indistinguishable (Fig. 5A). These results showed that PtsO and PtsP must both be present to modulate *lasI* expression in the absence of PtsN.

### PtsO represses *lasI* transcription through its conserved phosphorylation site

The requirement for both PtsP and PtsO in suppressing *lasI* transcription implied that PtsO phosphorylation, which depends on PtsP, might have a regulatory role. Thus, we considered the possibility that phosphorylated PtsO represses *lasI* expression. To test this hypothesis directly, we ectopically expressed *ptsO* from the neutral *attB* site in the chromosome of the ∆*ptsO-*∆*ptsN* mutant (where PtsP is present). In this mutant, introducing *ptsO* caused a small but significant decrease in *lasI* expression (Fig. 5C), which correlated with the stationary phase in time-course experiments (Fig. 5D; Fig. S6B). Next, we constructed and tested a *ptsO* allele in which the conserved phosphorylation site (His15) is substituted with an alanine. PtsO has a high degree of conservation across many bacterial species, and the His15 phosphorylation site is universally conserved across this family (34). Mutation of this residue to alanine disrupts PtsO-dependent gene regulation in the related species *Pseudomonas putida* (35). To test the hypothesis that the His15 residue of PtsO is important for it to repress *lasI*, we moved the PtsO^H15A^ gene into the chromosome of the ∆*ptsO-*∆*ptsN* strain and assessed activation from the *lasI* promoter using the P*_lasI_*_-_*gfp* reporter plasmid. We did not observe any decrease in transcription from the *lasI* promoter in the strain with the PtsO^H15A^ allele as we did for the strain with PtsO (Fig. 5C and D). There was also no significant effect on growth or PtsO levels in whole-cell lysates that could explain this result (Fig. S6). Although there are other potential explanations, such as misfolding of the PtsO^H15A^ protein, these results are consistent with the idea that PtsO represses *lasI* expression due to phosphorylation at its His15 residue.

In our experiments, the ability of PtsO to repress *lasI* transcription did not require PtsN, suggesting that PtsO repression is independent of its role in phosphotransfer to PtsN, the terminal acceptor. There are two other enzymes in *P. aeruginosa* with terminal phosphoacceptor activity analogous to that of PtsN; these are the carbohydrate PTS enzymes FruB and NagF. FruB can act as both a phosphodonor and a phosphoacceptor and, as mentioned above, is known to donate phosphate to PtsN in the absence of PtsO (7). However, it is not known whether FruB can accept phosphate from PtsO. Thus, we sought to test the hypothesis that FruB or NagF could serve as an alternative phospho-recipient for PtsO. We expected that if FruB or NagF are phosphorecipients for PtsO*,* then deleting *fruB* or *nagF* from the wild-type genome would increase *lasI* expression similar to deleting *ptsO*. However, deleting *fruB* and *nagF* had no measurable effects on *lasI* expression (Fig. S7). These results support the conclusion that NagF and FruB are not required for PtsO to repress transcription from the *lasI* promoter and that the regulatory mechanism of PtsO is independent of its role in transferring phosphate to downstream PTS enzymes.

### PtsO regulates a distinct subset of genes

**Fig 6:**
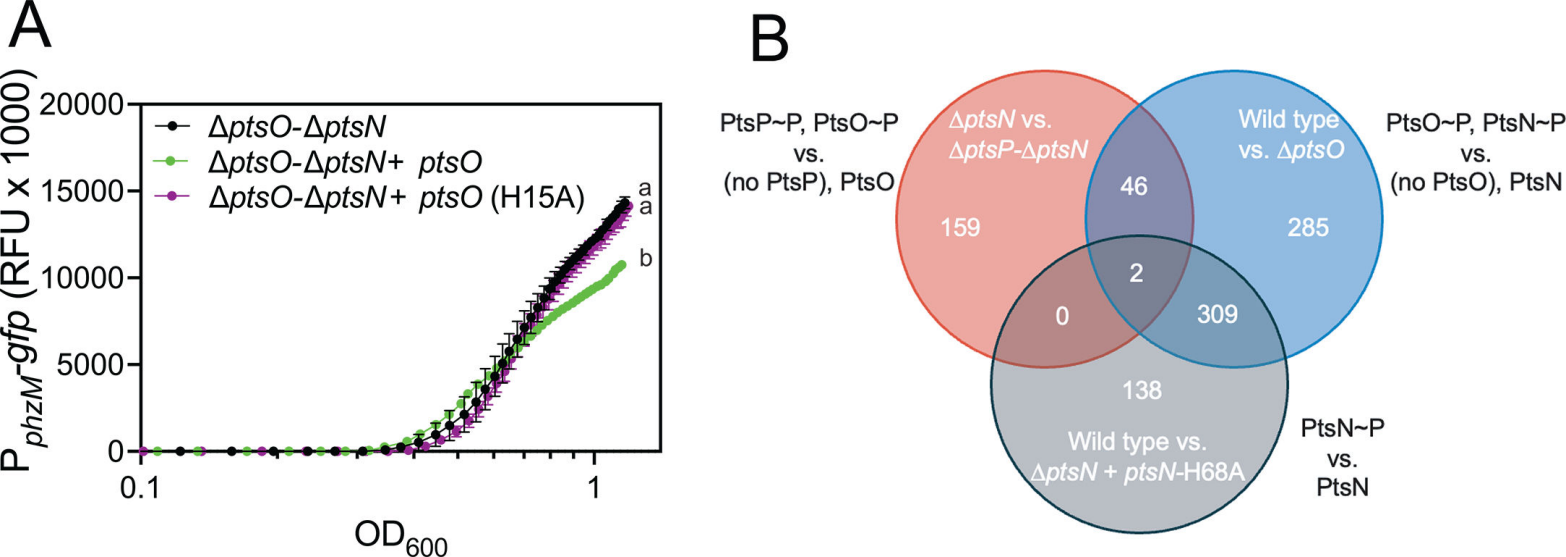
PtsO regulates a unique set of genes independent of PtsN. (**A**) PtsO decreases transcription from the *phzM* promoter due to its phosphorylation site. Fluorescence output was measured over a time course in 96-well plates using a BioTek plate reader. (**B**) Venn diagram showing overlap of genes downregulated in the first strain compared with the second strain listed, as indicated, with genes > 2-fold differentially expressed for each comparison. Genes for the wild type vs. ∆*ptsN +ptsN* H68A were from re-analyzing the data from the study of Underhill et al. (7).

PtsO is not known to modulate gene transcription independent of its role in phosphotransfer to PtsN in *P. aeruginosa*. Based on our above results with the *lasI* gene, we sought to determine if PtsO similarly regulates other genes by a PtsN-independent mechanism. First, we examined *phzM* and *hcnA* because of our finding that expression from these promoters was affected by the ∆*ptsP* mutation (Fig. 1). For this experiment, we introduced the P*_phzM-_gfp* and P*_hcnA_-gfp* reporter plasmids to the ∆*ptsO-*∆*ptsN* mutant with ectopically expressed PtsO, PtsO^H15A^, or neither, and compared fluorescence intensities over a time course. We observed a small but significant reduction of *phzM* reporter activity due to the expression of PtsO that was not observed with PtsO^H15A^ or with no PtsO (Fig. 6A), which was not explained by differences in growth (Fig. S8A). For *hcnA,* we observed no statistically significant differences between ∆*ptsO-*∆*ptsN* and the other two strains, but there was a significant difference between the PtsO and PtsO^H15A^ strains, albeit small (Fig. S9). These results support the idea that phosphorylated PtsO decreases transcription from the promoters of *phzM* and, to a much lesser degree, *hcnA*. We also assessed whether *phzM* transcription is regulated by PtsN. To test this, we introduced the P_*phzM*_-*gfp* reporter to the ∆*ptsN* strain with ectopically expressed PtsN, PtsN^H68A^, or neither. We found that PtsN^H68A^, but not PtsN, increased *phzM* expression >3-fold compared with wild type (Fig. S8B). Thus, both PtsO and PtsN modulate transcription from the *phzM* promoter as they do for the *lasI* promoter.

Our finding that PtsO regulates both *phzM* and *lasI* independent of PtsN led us to ask whether PtsO independently regulates a broader set of genes. To this end, we took advantage of previously conducted transcriptomic studies aimed at elucidating the PtsN regulon (7). This prior analysis included wild-type, ∆*ptsN,* and ∆*ptsP-*∆*ptsN* strains, and for this study, we added an unpublished Δ*ptsO* transcriptome that was collected and analyzed with the strains from the original experiment. These prior studies were carried out in a synthetic cystic fibrosis sputum medium, which was different from the lysogeny broth used for our reporter analyses; nevertheless, this approach provided an opportunity to determine whether PtsO has a distinct regulon from that of PtsN. Furthermore, the results were useful to examine whether the regulatory role of PtsO in the absence of PtsN extends to other conditions. We included genes regulated >2-fold to capture relatively small regulatory effects such as those we observed with *lasI*.

To determine the PtsO regulon, we first compared differentially expressed genes in wild-type and ∆*ptsO* strains to identify genes impacted by the presence of PtsO (presumed to be phosphorylated in the wild-type strain) vs. no PtsO. Note that PtsN phosphorylation also differs between these strains, as PtsN is unphosphorylated in Δ*ptsO* (7). We identified 642 genes that were downregulated in the wild type compared with ∆*ptsO*. We then compared differentially expressed genes in ∆*ptsN* and ∆*ptsP-*∆*ptsN* as another way to deduce genes regulated by PtsO; in this case, the strains were presumed to have different phosphorylation states of PtsO (phosphorylated in ∆*ptsN* vs. unphosphorylated in ∆*ptsP-*∆*ptsN*). The absence of PtsN in these strains eliminates any regulatory effects due to PtsN phosphorylation, but we note that the presence of PtsP differs between these strains. We identified 207 downregulated genes in ∆*ptsN* compared with ∆*ptsP-*∆*ptsN,* and more importantly, 48 genes downregulated in both the first and second strain comparisons (Fig. 6B). Because PtsO is phosphorylated in the first strain in both comparisons, this list of 48 genes includes genes downregulated by phosphorylated PtsO.

In principle, the 48 genes identified above could also include genes downregulated by both phosphorylated PtsP and PtsN, which also differ in the first and second comparisons, respectively. To this end, we included a third comparison that served to exclude genes downregulated by phosphorylated PtsN. This third comparison was of wild type and ∆*ptsN* complemented with the PtsN^H68A^ phosphorylation site mutant. In this comparison, we uncovered 449 genes downregulated by phosphorylated PtsN (Fig. 6B), with a majority (69%) of these genes also downregulated in the wild type vs. Δ*ptsO* comparison, presumably due to the difference in PtsN phosphorylation, which is common to both comparisons. Importantly, 48 of the 48 genes from our first two strain comparisons were excluded from those identified to be regulated by PtsN. These results lend confidence in these 48 genes as candidates for repression by phosphorylated PtsO (Table S4).

Among the 48 candidate PtsO-repressed genes, the most highly regulated were those encoding tRNAs (tRNA-Leu, tRNA-Glu, and tRNA-Gly), and the aminoglycoside-specific MexXY efflux pump. Notably, the *mexXY* genes were more strongly downregulated in ∆*ptsN* vs. ∆*ptsP*-∆*ptsN* (~45-fold) than in the wild type compared with ∆*ptsO* comparison (~3-fold), suggesting that these genes may have differing responses to different PTS^Ntr^ enzymes. Of note, neither *lasI* nor *phzM* were among this list of 48 genes; however, they may have been missed because the expression changes were relatively small (~3-fold in our reporter experiments) or because of the differences in growth conditions. Nevertheless, the results of our transcriptomic analysis support the idea that PtsO and PtsN can have independent effects on gene regulation.

## DISCUSSION

**Fig 7:**
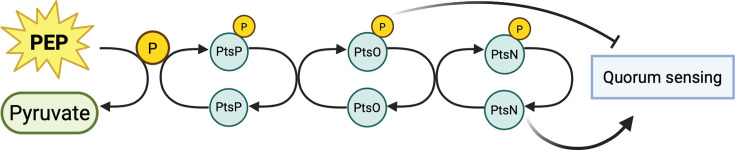
Model of PTS^Ntr^ regulation of quorum sensing. Transcription of several quorum-sensing genes, such as the signal synthase gene *lasI,* is suppressed by phosphorylated PtsO and activated by unphosphorylated PtsN. Deleting *ptsP* presumably blocks phosphorylation of both PtsO and PtsN, and both enzymes contribute to the observed increase in gene transcription.

In this study, we examined the regulatory link between PTS^Ntr^ and quorum sensing in *P. aeruginosa*. We identified several quorum sensing-regulated genes that are also regulated by PTS^Ntr^. By examining regulatory effects on *lasI,* the gene encoding the synthase of the LasR-specific signal, we delineated the role of each of the three PTS^Ntr^ enzymes in gene regulation and showed that modulating phosphorylation of either PtsO or PtsN is sufficient to impact *lasI* gene expression. Our results suggest a model where blocking phosphotransfer through the PTS^Ntr^ mediates effects through PtsN and PtsO and that the PtsO effects are independent of its role in phosphotransfer to PtsN (Fig. 7). These data support the notion that the constituent enzymes of PTS^Ntr^ have roles beyond the classic phosphotransfer cascade. Consistent with this idea, we also identified a PtsO-specific regulon that is distinct from that of PtsN.

Collectively, our study adds to the growing body of knowledge of the PTS^Ntr^ in *P. aeruginosa* and provides new information on its connection to quorum sensing regulation. In the absence of PtsP, additive effects of both PtsN-dependent and PtsN-independent regulation increase *lasI* expression (Fig. 2A and 3C). The increase in *lasI* expression causes a corollary increase in the LasI-dependent quorum sensing signal, 3OC12-HSL (Fig. 2), although in the conditions of our experiments, we did not observe that the increase in 3OC12-HSL causes substantial activation of other quorum sensing genes (e.g., *lasB*, Fig. 1). Most likely, these genes are already maximally activated by the level of 3OC12-HSL produced by the wild-type strain. Several genes that are regulated (e.g., *lasI* and *phzM*) may be the targets of a common transcription regulator that is influenced by the PTS^Ntr^ enzymes, which functions along with LasR to regulate their expression, similar to an “AND” logic gate that results in additive effects from two variables. There are other possible explanations, such as the PTS^Ntr^ somehow increasing gene activation by LasR. The mechanism by which the PTS^Ntr^ interacts with the Las quorum-sensing system warrants further inquiry.

A major finding of our study is that PtsO has distinct regulatory effects that are independent of its role in phosphotransfer to PtsN. An alternative possibility is that there are effects of PtsP that depend on blocking its ability to transfer phosphate to PtsO. Either way, the results support the idea that PtsP and PtsO have functions that are independent of the classic phosphotransfer cascade. There is evidence from other species that the PTS^Ntr^ enzymes can function independent from each other; for example, in *E. coli,* PtsO regulates lipid A biosynthesis (36, 37) and envelope stress responses (38). There may be a benefit of having distinct regulatory outputs of PTS^Ntr^, such as an increased range or sensitivity to different inputs. For example, PtsN can be phosphorylated by the PTS^Fru^ enzyme FruB in both *P. aeruginosa* (7) and *P. putida* (39), and in principle, FruB phosphorylation of PtsN might lead to differential phosphorylation states of PtsN and PtsO. This type of regulation might enable the cells to respond independently to different inputs or fine-tune regulatory responses.

We found it interesting that deleting the GAF domain does not phenocopy the ∆*ptsP* mutation in our *lasI* reporter studies (Fig. 3B). These results are consistent with prior observations of biofilm formation (22), where the GAF-deficient PtsP variant also did not phenocopy the ∆*ptsP* mutant. The GAF domain has an established role in phosphotransfer to PtsN (7), but the results of our *lasI* regulation and biofilm studies suggest that we do not yet fully understand the function(s) of PtsP. It is formally possible that the GAF domain has condition-specific effects and is not required for PtsP-mediated phosphotransfer to the downstream PTS^Ntr^ enzymes in some conditions. More likely, PtsP may play an as-yet unknown role in downstream gene regulation that is independent of its role in phosphotransfer to the downstream PTS^Ntr^ enzymes. For example, the GAF-deficient PtsP variant may block phosphotransfer to PtsN (which would normally increase *lasI* expression) while at the same time functioning to reduce *lasI* expression through an alternative, PtsN-independent pathway. In any case, these findings warrant further inquiry into how PtsP influences gene regulation independently of its effects on PtsN.

In our transcriptomic analyses, the presence of phosphorylated PtsO was associated with suppressed synthesis of tRNAs that can be charged with lysine, leucine, selenocysteine, glutamate, and glycine. These results suggest phosphorylated PtsO might alter the synthesis of certain proteins based on their amino acid composition. The depletion of certain tRNAs could also slow down translation or cause ribosome stalling, which could serve to induce certain stress responses (e.g., the stringent response). It is notable that the *mexXY* genes, which are known to be induced by ribosome stalling (40–42), were also downregulated by phosphorylated PtsO, which could be due to the effects on translation. These genes were also more strongly repressed in the ∆*ptsN* vs. ∆*ptsP-*∆*ptsN* comparison than in the wild type vs. ∆*ptsO* comparison, suggesting that *mexXY* may have an additional level of repression by PtsP, which differs in the first comparison and not in the second. Further studies of the link between the PTS^Ntr^ and MexXY, antibiotic resistance, or both are needed to better understand this connection.

To date, few direct PTS^Ntr^ targets have been identified. The protein targets of the PTS^Ntr^ phosphoenzymes are assumed to be regulated through protein-protein interactions. In *E. coli,* TrkA, a K^+^ transporter, was identified as the direct target of PtsN (15), and the *P. putida* PtsN directly targets the PDH enzyme complex, which converts pyruvate to acetyl-CoA in the TCA cycle (43, 44). To our knowledge, no direct targets have been studied in *P. aeruginosa*. Nonetheless, our studies of quorum sensing support the idea that PtsO and PtsN may have unique direct targets that each regulate different downstream genes.

## MATERIALS AND METHODS

### Bacterial culture conditions and reagents

Bacteria were routinely grown in Lysogeny Broth (LB) (if *E. coli*) or LB buffered to pH 7 with 50 mM 3-(morpholino)-propanesulfonic acid (MOPS) (if *P. aeruginosa*), or on LB agar (LBA; 1.5% wt/vol Bacto-Agar; for both *E. coli* and *P. aeruginosa*), unless otherwise specified. All *P. aeruginosa* broth cultures were grown in 18 mm test tubes (for 2 mL cultures) at 37°C with shaking at 250 rpm, 125 mL baffled flasks (for 10 mL cultures), 250 mL baffled flasks (for 50 mL cultures), or in a 96-well plate using 200 µL growth media. For *E. coli*, 10–20 µg mL^−1^ gentamicin (depending on the strain), 10 µg mL^−1^ tetracycline, and 100 µg mL^−1^ ampicillin were used. For *P. aeruginosa*, 50–200 µg mL^−1^ gentamicin (on LBA), 15 µg mL^−1^ gentamicin (in LB broth), and 200 µg mL^−1^ tetracycline were used. The CTX-2-P_ara_-*lasI* strains were grown in either 0% or 0.5% arabinose to induce plasmid expression. N-3-oxo-dodecanoyl-L-homoserine lactone (3OC12-HSL) was purchased from Cayman Chemicals (Ann Arbor, MI, USA), dissolved in acidified ethyl acetate (ethyl acetate mixed with 0.1  mL^−1^glacial acetic acid) (45), and added to culture tubes and dried using nitrogen gas prior to adding cultures. Genomic or plasmid DNA was extracted using Qiagen Puregene Core A kit (Hilden, Germany) or IBI Scientific plasmid purification mini-prep kit (IA, USA), whereas PCR products were purified using IBI Scientific PCR clean-up/gel extraction kits, according to the manufacturer’s protocol. Gentamicin antibiotics were purchased from GoldBio (MO, USA), tetracycline was purchased from Fisher Scientific (PA, USA), and ampicillin was purchased from Sigma Aldrich (MO, USA). To measure luminescence (β-galactosidase) activity, the Galacto-Light Reaction Tropix kit from ThermoFisher Scientific (PA, USA) was used.

### Bacterial strains and strain construction

All bacterial strains and plasmids used in this study are listed in Tables S2 and S3. *P. aeruginosa* strain UCBPP-PA14 (‘PA14’) (46) and PA14 derivatives were used for these studies. The allelic exchange was used to make markerless deletions in specific loci of *P. aeruginosa* PA14 as described elsewhere (47). For allelic exchange, DNA fragments carrying the mutated or deleted gene allele plus 500 bp flanking DNA were synthesized by GenScript and inserted into the pEXG2 suicide vector. During the process of allelic exchange, the plasmids were moved to *P. aeruginosa* by conjugation using an *E. coli* donor strain and transformants were selected on Pseudomonas Isolation Agar (PIA) using gentamicin (200 µg mL^−1^) and counterselected on NaCl-free LB agar containing 15% sucrose. Putative mutants were verified through antibiotic sensitivity tests and gene-targeted Sanger sequencing. CTX plasmids were also moved to *P. aeruginosa* strains via conjugation from an *E. coli* donor strain. DNA fragments from CTX that incorporated at the neutral chromosomal *attB* locus were selected on PIA-Tetracycline (200 µg mL^−1^) and verified by PCR (22, 48). All replicating plasmids were introduced to *P. aeruginosa* via electroporation (30), selected on LB agar using gentamicin at 50-200 µg mL^−1^, and routinely grown with gentamicin (50 µg mL^−1^ for agar and 15 µg mL^−1^ for broth) for plasmid maintenance.

### Reporter activity measurements

The influence of the PTS^Ntr^ system on *lasI, phzM, rhlA, lasB, rsaL,* and *hcnA* gene expression levels in *P. aeruginosa* was measured using transcriptional reporter plasmids with each gene promoter fused to the promoterless *gfp* reporter in the pPROBE plasmid. Each plasmid was moved to *P. aeruginosa* by electroporation, and single transformant colonies were used to inoculate 2 mL LB-MOPS with gentamicin for selection to start the experiments. For end-point measurements of activity, the cells were grown for 18 h, washed with phosphate-buffered saline (PBS), and suspended in PBS, and fluorescence and optical density were measured using a BioTek Synergy 2 plate reader. For time-course measurements, overnight cultures were diluted 1:100 in fresh medium and grown to an optical density at 600 nm (OD_600_) of ~0.1. This culture was then diluted to an OD_600_ of ~0.004 and dispensed in a black 96-well clear flat-bottom plate with 200 µL per well. Plates were incubated with double orbital shaking at 37°C in a Biotek Synergy H1 plate reader with OD_600_ and GFP fluorescence (excitation 485 nm, emission 528  nm) measured every 15 min for 14 h. To account for background, the empty vector pPROBE P*_empty_*-gfp was also measured and subtracted from the measured values of each reporter strain. The final fluorescence values were plotted with respect to OD_600_ using GraphPad Prism.

### Quorum sensing signal measurements

We used a bioassay to measure 3OC12-HSL produced by different strains of *P. aeruginosa*. To prepare the samples for the analysis, we used 5 mL cultures grown 18 h in LB-MOPS. The cells were removed from the culture fluid by centrifugation, the culture fluid was extracted twice with acidified ethyl acetate, and the ethyl acetate fraction was evaporated to dryness under a stream of nitrogen gas. The residue was dissolved in 0.5 mL acidiﬁed ethyl acetate, and the ethyl acetate solutions were used in bioassay. For the bioassay, we used strain *E. coli* DH5a pSC11 pJ105L-LasR with P*_lasI_-lacZ* and *lasR* on different plasmids (30). Details of the bioassay procedure have been described elsewhere. Briefly, overnight *E. coli* cultures grown in LB-MOPS were diluted 1:100 into fresh LB-MOPS and grown to an OD_600_ of ~0.2–0.3 prior to adding arabinose for induction of *lasR*. The culture was then grown to an OD_600_ of ~0.5–0.6, and aliquots (0.5 mL) were dispensed into 2 mL Eppendorf tubes with evaporated culture fluid extracts, synthetic 3OC12-HSL standards or no signal and grown an additional 3 h. β-galactosidase was detected using the Galacto-light Reaction Tropix Kit (Thermo Fisher) and measured using a Biotek Synergy 2 plate reader. A standard curve was generated from the synthetic signal samples, and signal levels in the culture fluid extracts were determined by comparing them with the standard curve.

### RNA extraction and sequencing for transcriptomics

RNA extraction and sequencing were as described previously (7). Briefly, cultures were grown with shaking at 37°C in a synthetic cystic fibrosis medium (SCFM2) and harvested in logarithmic phase at an OD_600_ of 0.3, and RNA was extracted according to the Monarch RNA extraction kit (New England Biolabs, MA, USA). rRNA was depleted using the Illumina RiboZero kit (Illumina, CA, USA), and the samples were sequenced by 150 bp paired-end Illumina sequencing at the University of Oklahoma Health Sciences Center core facility in Oklahoma City, OK, USA. Sequence mapping and analysis were performed at the Oklahoma University Health Sciences Center Laboratory for Molecular Biology and Cytometry Research using CLC software. Genes were classified as differentially expressed in pair-wise comparisons based on the FDR-adjusted *P*-value < 0.001 and log_2_ fold-change values > 1, and a summary of the overlap in differentially expressed genes between pair-wise comparisons was generated with the R package ggVennDiagram v 1.5.2 in R v 4.4.1 (49, 50). The NCBI accession number for the deposited RNAseq data is GSE297711.

## SUPPLEMENTAL MATERIAL

Supplemental material jb.00048-25-s0001.pdf Supplemental methods, Fig. S1 to S9, Tables S1 to S3, and supplemental references.

Table S4 jb.00048-25-s0002.xlsx Gene annotation.

ASM does not own the copyrights to Supplemental Material that may be linked to, or accessed through, an article. The authors have granted ASM a non-exclusive, world-wide license to publish the Supplemental Material files. Please contact the corresponding author directly for reuse.
