# Supplementary material for: Quorum sensing regulation by the nitrogen phosphotransferase system in Pseudomonas aeruginosa
Source: J Bacteriol. 2025 Aug 1;207(8):e00048-25. doi: 10.1128/jb.00048-25 (PMC12369347; doi:10.1128/jb.00048-25)
Supplement: Supplemental material — Supplemental methods, Fig. S1 to S9, Tables S1 to S3, and supplemental references. [file jb.00048-25-s0001.pdf]

SUPPLEMENTAL MATERIAL FOR:

Quorum sensing regulation by the nitrogen phosphotransferase system in *Pseudomonas aeruginosa*

AUTHORS:

Samalee Banerjee,[a] Nicole Smalley,[b] Pradtahna Saenjamsai,[a] Anthony R. Fehr,[a] Ajai A. Dandekar,[b] Matthew Cabeen,[c] Josephine R. Chandler[a]

[a]Department of Molecular Biosciences, University of Kansas, Lawrence, KS.

[b]Department of Microbiology, University of Washington, Seattle, WA.

[c]Department of Microbiology and Molecular Genetics, Oklahoma State University, Stillwater, Oklahoma.

Supplementary methods

Quantitative PCR. RNA was harvested from LB-grown stationary-phase *P. aeruginosa* cells (OD_{600} of ~4) using methods described previously (1). Droplet digital PCR was performed on Bio-Rad's QX200 Droplet Digital PCR System using Eva Green Supermix. Each reaction used 1 ng μl−1 of cDNA template, 0.25 μM primer, 10 μl Eva Green Supermix, and 8 μl H2O in a 20-μl volume. After generating 40 μl of oil droplets, 40 rounds of PCR were conducted using the following cycling conditions: 94°C for 20 sec, 58°C for 20 sec, and 72°C for 20 sec. Absolute transcript levels were determined using the Bio-Rad QuantaSoft Software. In all cases a no template control was run with no detectable transcripts. The proline biosynthesis gene *proC* was used as a reference gene and the results are reported as the calculated transcript amount of a given gene per calculated *proC* transcript.

Western blot. Cells for Western blot were grown in test tubes at 37°C with shaking for 18hrs, at which point the cell densities were measured at an optical density of 600 nm (OD_{600}) and determined to be equivalent within 5%. Samples (1 ml) were then centrifuged, and the cell pellets were washed twice with cold 1X TBS (2 0mM Tris-HCl, pH 7.5, 150 mM NaCl), and the washed cell pellets were subjected to a cycle of freeze-thaw at -80°C. The cell pellet was then dissolved in 50 mM Tris buffer, using 50 μL of Tris buffer (50 mM Tris-HCl, pH 7.5), and lysed by adding 100 μL loading buffer (60 mM Tris pH 6.8, 2% sodium dodecyl sulfate [SDS], 10% Glycerol, 0.01% bromophenol blue) supplemented with 1X Protease Inhibitor Complex (PIC) and Phosphatase Inhibitor Complex (PhIC), 1% Phenylmethylsulfonyl fluoride (PMSF) and 1% β-Mercaptoethanol (BME) and this was treated with 1 μl Universal Nuclease for 1hr at 37°C. The samples were heated at 95°C for 5-10 minutes, centrifuged at 16,000g for 20mins and

supernatant was collected. The supernatant was further diluted by sample buffer (1:5 ratio) in 2X SDS sample buffer and equal amounts of sample for each strain was loaded. For Western blots, primary antibody (DYKDDDDK Tag Antibody, mAb, Mouse, from Genscript) and secondary antibody (Goat anti-mouse, IRDye 680RD, from LICOR) was used and membranes were imaged using a LICOR Odyssey M imager.

Supplementary Figures

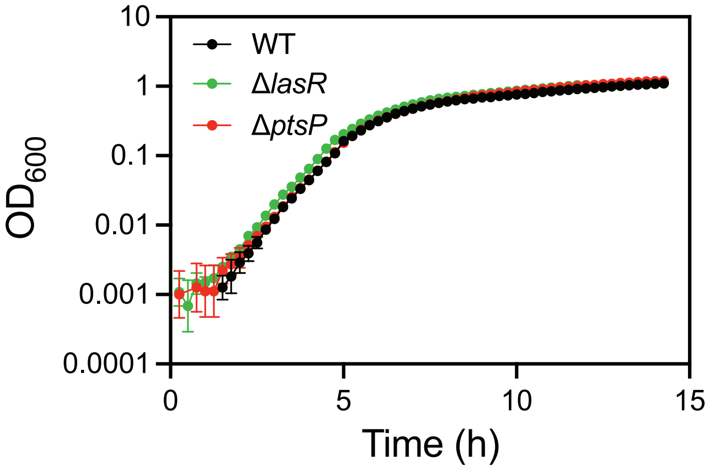

Figure S1. Growth of strains in experiments presented in Fig. 1. Data show growth of PA14 strains transformed with the pP$_{lasI}$-*gfp* plasmid; all other reporter plasmids showed similar results. OD_{600} refers to the optical density at 600 nm, which was measured using a BioTek plate reader at the same time as the fluorescence measurements shown in Fig. 1. Data points are the means of three biological replicates, and the error bars represent standard deviation.

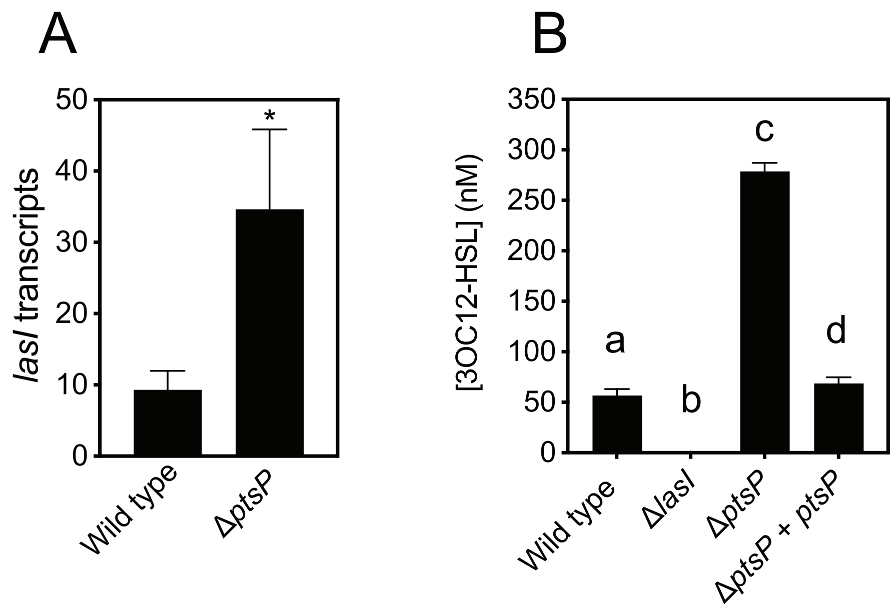

Fig. S2. Δ*ptsP*-dependent changes in (A) *lasI* transcripts and (b) 3OC12-HSL levels. For A, stationary-phase cells were harvested after 8 h growth in LB-MOPS, which corresponds with an optical density at 600 nm (OD_{600}) of ~4 where cultures had just entered stationary phase. Note that the OD_{600} measurements vary from that of Fig. S1 because they were taken using a benchtop spectrophotometer (vs. a plate reader for Fig. S1). Results show *lasI* transcripts normalized to the housekeeping gene *proC.* Statistical analysis was by t-test compared with wild type: *, $p \leq 0.02$. For B, Stationary-phase cells were extracted after 18 h growth in LB-MOPS and the concentration of 3OC12-HSL was measured using a bioassay and determined by comparison with a standard curve using synthetic 3OC12-HSL. Statistical significance was by one-way ANOVA with Tukey's multiple comparisons *post-hoc* analysis; different letters indicate $p<0.05$ and same letters indicate $p>0.05$. For both panels, values represent the average of three independent experiments and the vertical bars represent the standard deviation.

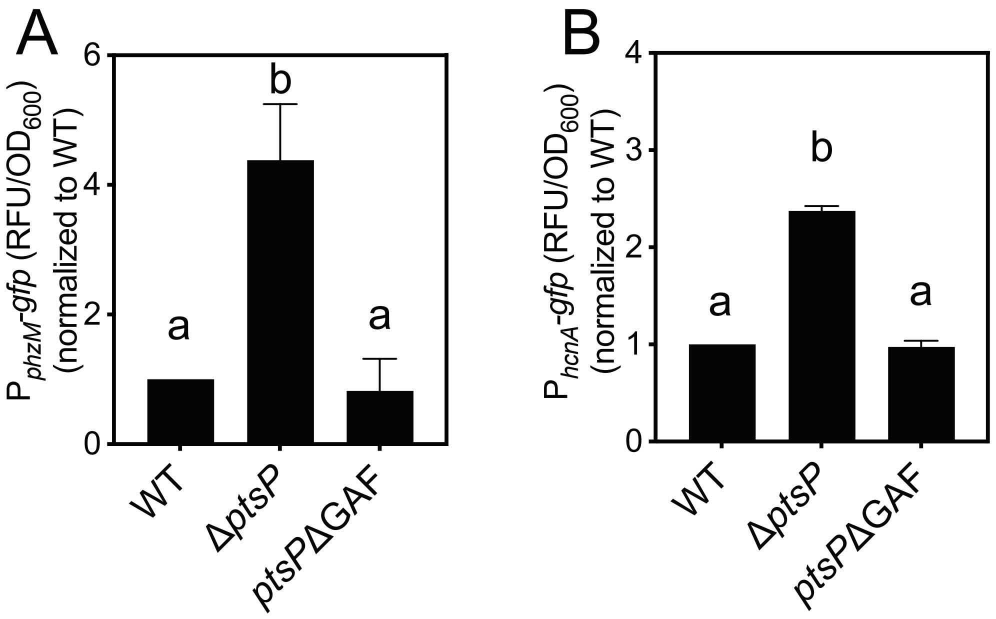

Figure S3. The GAF domain is dispensable for PtsP regulation of transcription from the *phzM* (A) and *hcnA* (B) promoters. Transcription was monitored as GFP fluorescence in cells transformed with the pP$_{phzM}$-*gfp* or pP$_{hcnA}$-*gfp* reporter plasmids. Data shown are growth-adjusted fluorescence after 18 h of growth. Data are means of three biological replicates, and vertical bars represent standard deviation. Statistical significance in comparison to wild type was assessed by one-way ANOVA using Tukey's multiple comparisons *post-hoc* analysis; different letters indicate comparisons where $p<0.001$ and the same letters indicate comparisons where $p>0.05$.

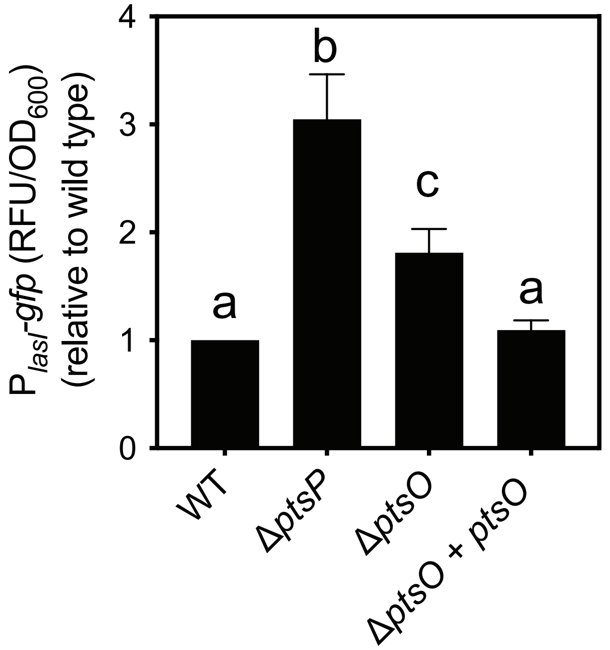

Figure S4. Δ*ptsO* complementation. Transcription from the *lasI* promoter was monitored as GFP fluorescence in cells transformed with the pP*lasI*-*gfp* reporter plasmid. Strains carried *ptsO* or the empty CTX cassette in the neutral *attB* site in the genome. Fluorescence was obtained after overnight growth and is normalized to OD_{600} and shown as the fold change compared with wild type. Data are means of three biological replicates, and the vertical bars represent the standard deviation. Statistical significance was by one-way ANOVA using Tukey's multiple comparisons *post-hoc* analysis; different letters indicate comparisons where $p<0.05$ and the same letters indicate comparisons where $p>0.05$.

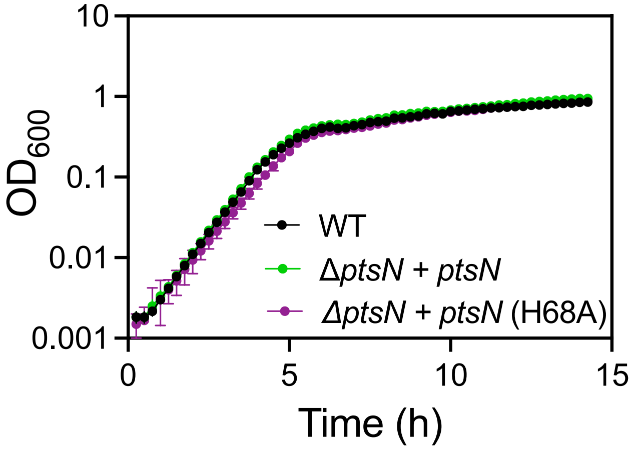

Figure S5. Growth of *ptsN* mutants. Growth curves for experiments presented in Fig. 4B in the main text. Strains carried the pP$_{lasI}$-*gfp* reporter plasmid and *ptsN, ptsN* (H68A) or an empty cassette inserted at the neutral *attB* site in the chromosome. Measurements were taken on a Biotek plate reader at the same time as the fluorescence measurements shown in Fig. 4B. Data points are the means of three biological replicates, and the vertical bars represent standard deviation.

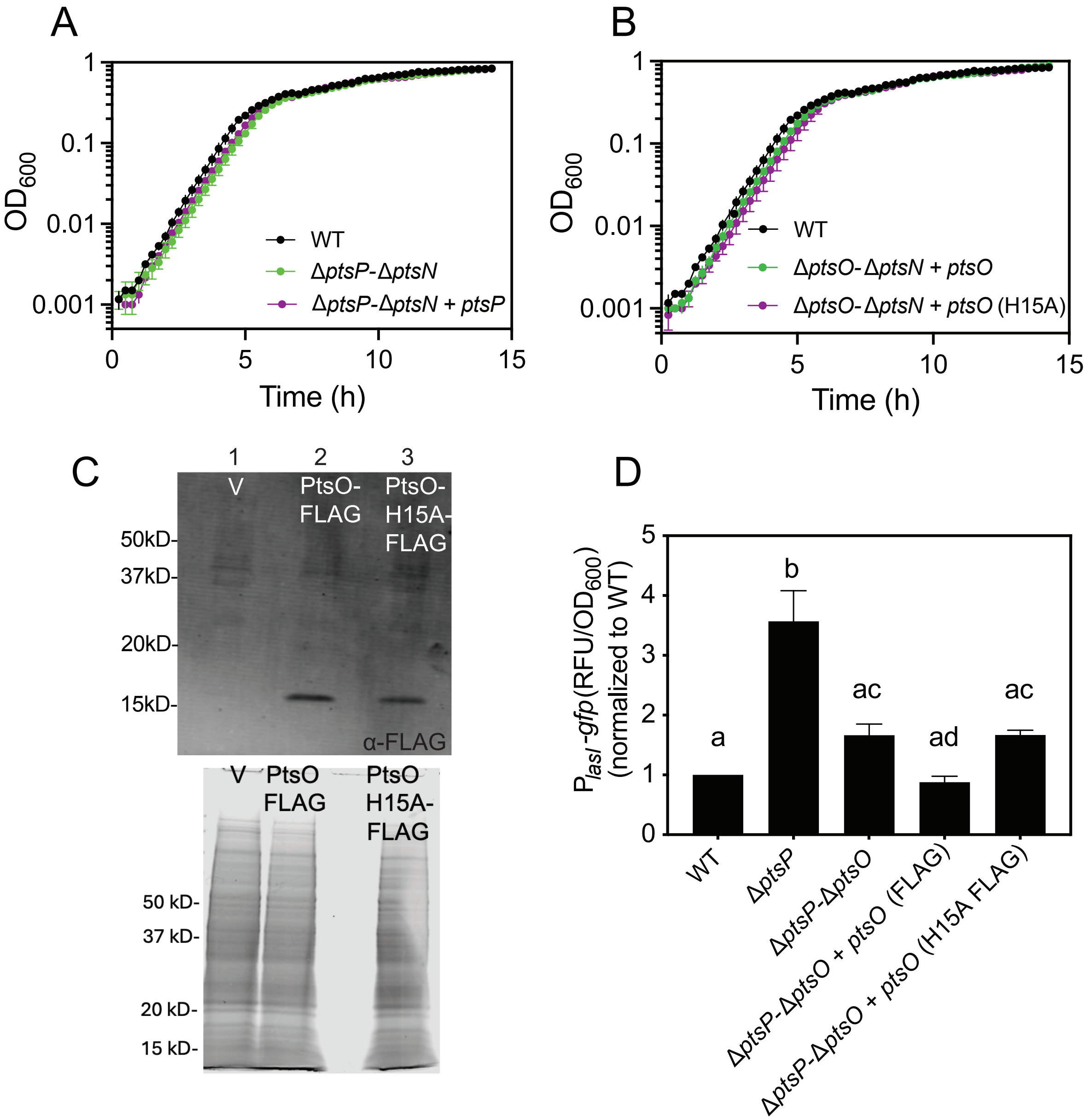

Fig. S6. Effects of PtsO and PtsO H15A on growth and PtsO levels. (A) Growth of strains in experiments presented in Figs. 5B (panel A) and 5D (panel B). All strains carried the CTX vector inserted into the neutral *attB* site in the genome, which carried either a gene encoding PtsP, PtsO, PtsO H15A, FLAG-tagged PtsO, FLAG-tagged PtsO H15A, or with no gene. Strains in panels A, B and D also carried the pP$_{lasI}$-*gfp* plasmid. For panels A and B, optical density at 600 nm (OD_{600}) was measured over a time course in 96-well plates using a BioTek plate reader at the same time as the fluorescence measurements shown in Figs. 5B and 5D. For panel C, the top

image is of a Western blot using anti-FLAG antibody as a probe. The lanes are of wild type (C), wild type encoding FLAG-tagged PtsO from the integrated CTX vector (PtsO-FLAG), or wild type with the FLAG-tagged PtsO H15A allele (PtsO H15A-FLAG). The bottom image is of a Coomassie-stained SDS-PAGE gel loaded with the same whole-cell lysates and run side-by-side with the Western blot, which was run as a loading control. For panel D, the effects of the FLAG tag on PtsO regulation of *lasI* was determined by measuring activation of the P_{lasI}-*gfp* reporter. Statistical significance was by one-way ANOVA using Tukey's multiple comparisons *post-hoc* analysis; different letters indicate comparisons where $p<0.05$ and the same letters indicate comparisons where $p>0.05$. Results in panels A, B and D show the means of three biological replicates, and vertical bars show the standard deviation.

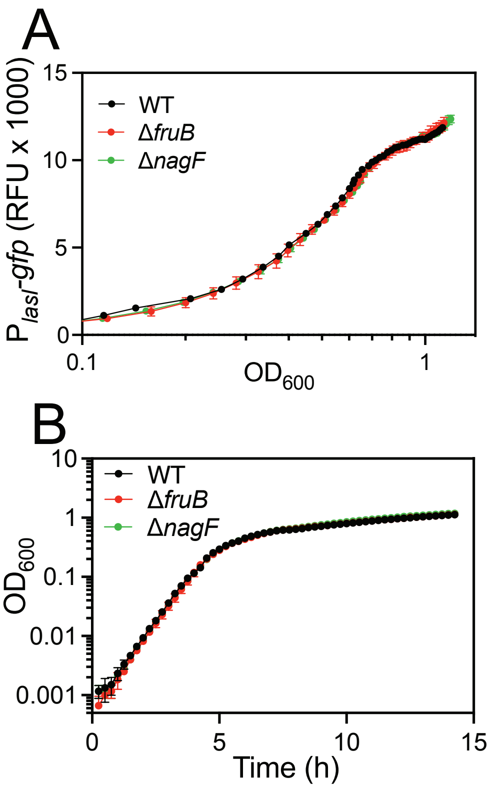

Fig. S7. *lasI* expression levels in strains with deletions of the carbohydrate phosphotransferase genes *nagF* and *fruB.* (A) Transcription from the *lasI* promoter was monitored as GFP fluorescence in cells transformed with the pP$_{lasI}$-*gfp* reporter plasmid. Fluorescence output measured over a time course in 96-well plates using a BioTek plate reader. There were no statistically significant differences between strains using the growth-adjusted fluorescence values from the final time points by one-way ANOVA using Tukey's multiple comparisons *post-hoc* analysis. (B) Growth of strains in experiments presented in panel A. Data points are the means of three biological replicates, and the vertical bars represent standard deviation. Optical density at 600 nm (OD_{600}) was measured over a time course in 96-well plates using a BioTek plate reader at the same time as the fluorescence measurements shown in panel A.

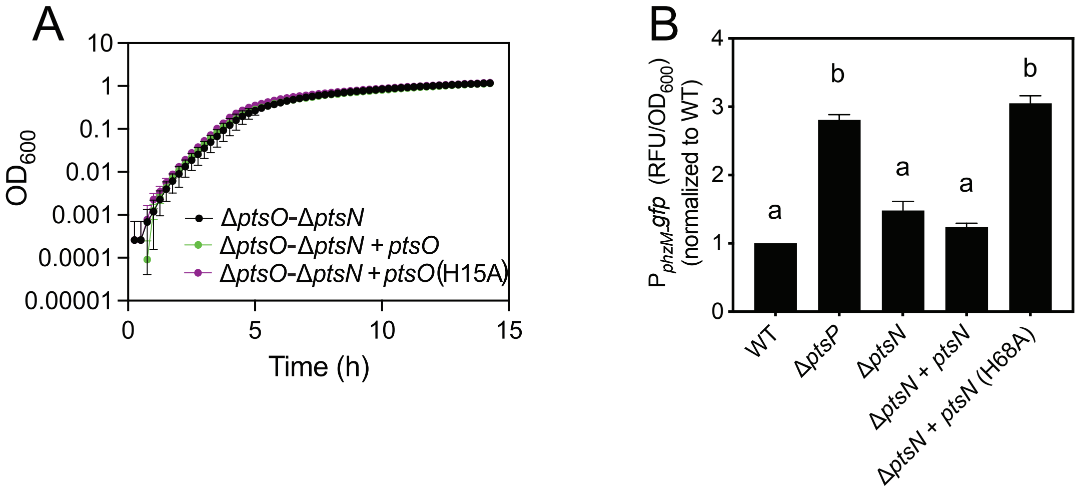

Fig. S8. The effects of PtsO and PtsN on *phzM* regulation. (A) Growth of strains in experiment presented in Fig. 6A in the main text, which is related to PtsO regulation of transcription from the *phzM* promoter. Optical density at 600 nm (OD_{600}) was measured over a time course in 96-well plates using a BioTek plate reader Optical density at 600 nm (OD_{600}) at the same time as the fluorescence measurements shown in Fig. 6A. (B) Transcription from the *phzM* promoter was measured using the pP$_{phzM}$-*gfp* plasmid. Fluorescence was measured after overnight growth and normalized to OD_{600}. Statistical significance was by one-way ANOVA using Tukey's multiple comparisons *post-hoc* analysis; different letters indicate comparisons where $p<0.05$ and the same letters indicate comparisons where $p>0.05$. For both panels, all strains had the chromosomally integrated CTX cassette, CTX plus the wild type *ptsO,* or CTX plus the *ptsO* allele encoding the H15A variant (*ptsO* (H15A)). Results show the means of three biological replicates, and vertical bars show the standard deviation.

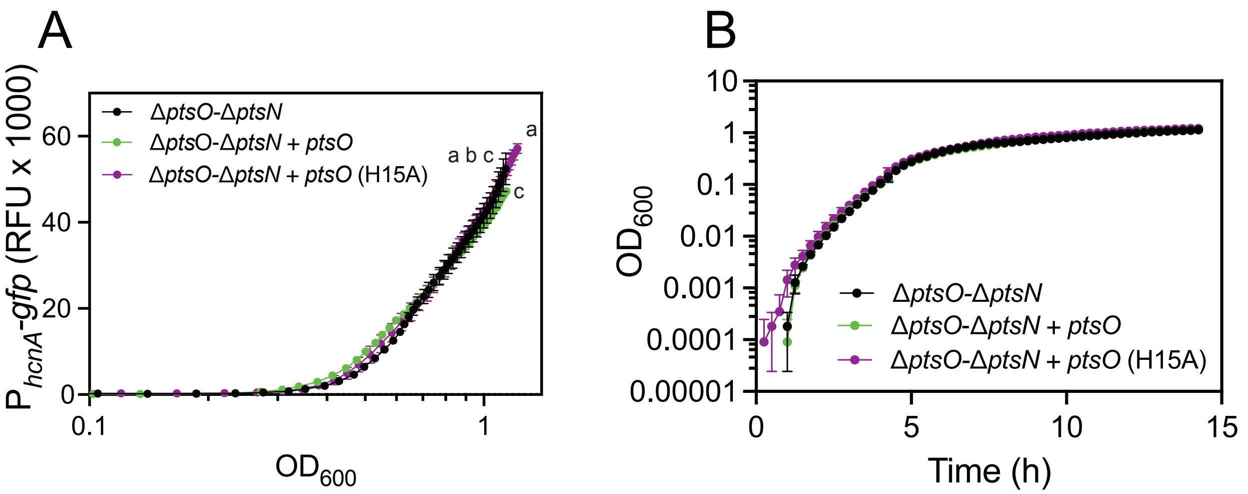

Fig. S9. The effects of PtsO on *hcnA* expression. (A) Transcription from the *hcnA* promoter was monitored as GFP fluorescence in cells transformed with the pP$_{phzM}$-*gfp* plasmid. To determine statistical significance we used one-way ANOVA using Tukey's multiple comparisons *post-hoc* analysis for the OD-adjusted fluorescence values of the final time point. Δ*ptsO*-Δ*ptsN* was not significantly different from the other two strains, but the strain with *ptsO* was significantly different from that of *ptsO* encoding the H15A variant ($p<0.03$). (B) Growth of strains in experiments presented in panel A. For both panels, strains carried a chromosomally integrated CTX-1 cassette (CTX), CTX plus the wild type *ptsO,* or CTX plus the H15A variant of PtsO (*ptsO* (H15A)), and fluorescence output and OD_{600} were measured over a time course in 96-well plates using a BioTek plate reader at the same time as the fluorescence measurements shown in panel A. Results are the means of three biological replicates, and the vertical bars represent standard deviation.

Supplemental Tables

Table S1. Summary of *lasI* reporter data

**Fig.**	**Strain**	***lasI-gfp* reporter activation**
**3**	Wild type	+
	Δ*ptsP*	+++
	Δ*ptsO*	++
	Δ*ptsN*	+
	Δ*ptsP*-Δ*ptsN*	++
	Δ*ptsP*-Δ*ptsO*-Δ*ptsN*	++
**4**	Δ*ptsN* + *ptsN*	+
	Δ*ptsN* + *ptsN* (H68A)	++
	Δ*ptsP*-Δ*ptsO*-Δ*ptsN* + *ptsN*	++
	Δ*ptsP*-Δ*ptsO*-Δ*ptsN* + *ptsN* (H68A)	+++
	Δ*ptsP*-Δ*ptsN* + *ptsN*	+++
	Δ*ptsP*-Δ*ptsN* + *ptsN* (H68A)	+++
**5**	Δ*ptsP*-Δ*ptsO*-Δ*ptsN* + *ptsP*	++
	Δ*ptsP*-Δ*ptsN* + *ptsP*	+
	Δ*ptsO*-Δ*ptsN*	++
	Δ*ptsO*-Δ*ptsN* + *ptsO*	+
	Δ*ptsO*-Δ*ptsN* + *ptsO* (H15A)	++

Table S2. Bacterial trains used in this study.

**Strains**	**Relevant properties**	**Reference or source**
P. aeruginosa **strains**		
**UCBPP- PA14 (PA14)**	Ancestral wild type	(2)
**PA14 Δ*ptsP***	PA14 with a deletion of *ptsP*	(3)
**PA14 Δ*lasR***	PA14 with a deletion of *lasR*	This study
**PA14 Δ*lasI***	PA14 with a deletion of *lasl*	(3)
**PA14 Δ*ptsO***	PA14 with a deletion of *ptsO*	(4)
**PA14 Δ*ptsN***	PA14 with a deletion of *ptsN*	(4)
**PA14 Δ*ptsP*-Δ*ptsO***	PA14 Δ*ptsO* with a deletion of *ptsP*	This study
**PA14 Δ*ptsP*-Δ*ptsN***	PA14 Δ*ptsN* with a deletion of *ptsP*	This study
**PA14 Δ*ptsP*-Δ*ptsO*-Δ*ptsN***	PA14Δ*ptsP*Δ*ptsN* with a deletion of *ptsO*	This study
**PA14 Δ*lasI*-Δ*ptsP***	PA14 Δ*lasI* with a deletion of *ptsP*	This study
**PA14 *ptsP*ΔGAF**	PA14 in which the coding sequence of the PtsP GAF domain has been deleted	(4)
**PA14 Δ*ptsO*-Δ*ptsN***	PA14 with *ptsO* and *ptsN* gene deletions	(5)
Escherichia coli **strains**		
**DH5α**	F^- ϕ80*lacZ*ΔM15 Δ(*lacZYA-argF*)*U169* *hsdR17*(r_K^- m_K^+) *recA1 endA1 phoA supE44 thi-1 gyrA96 relA1* λ^-	Invitrogen
**S17-1**	*recA pro hsdR* RP4-2-Tc::Mu-km::Tn*7*	(6)
**SM10**	*thi thr leu tonA lacY supE recA*::RP4-2-Tc::Mu Km λ*pir*	(6)
**DH5α pSC11 pJ105L-LasR**	3OC12-HSL-responsive bioassay strain	(7)

Table S3. Plasmids used in this study.

**Plasmid**	**Relevant properties**	**Reference or source**
pPROBE-GT	Broad-host-range pVS1/p15a GFP reporter; Gm^R	(8)
pCTX-1	Mini-CTX, *P. aeruginosa* integrative plasmid; Tet^R	(9)
pCTX-2	Mini-CTX with arabinose-inducible promoter; Tet^R	(10)
pEXG2	Suicide plasmid for allelic replacement; Gm^R	(11)
pBS351:P_{lasI}-*gfp*	Encodes −1 through −501 relatives to the start of *lasI*	(7)
pPROBE-P_{rsaL}-*gfp*	Encodes +103 to −290 relatives to the start of *rsaL*	(12)
pPROBE-P_{phzM}-*gfp*	Encodes +20 to −350 relatives to the start of *phzM*	This study
pPROBE-P_{rhlA} -*gfp*	Encodes +1 through −501 relatives to the start of *rhlA*	(7)
pPROBE-P_{lasB} -*gfp*	Encodes +217 to −290 relatives to the start of *lasB*	(13)
pPROBE-P_{hcnA} -*gfp*	Encodes +1 through −500 relatives to the start of *hcnA*	This study
pCTX1-*ptsP*	CTX-1 containing the *ptsP* gene, Tet^r	(4)
pSW196-RBS-*lasI*	Mini-CTX2 with P_{ara}*BAD* promoter; Tet^r	(14)
pCTX-1-Pop-*ptsN*	CTX-1 with *ptsN* fused to the *rpoN* operon promoter	(5)
pCTX-1-Pop-*ptsN* (H68A)	pCTX-1-Pop-*ptsN* with His 68 codon mutated to Ala	(5)
pCTX1-*ptsO*	PA14 *ptsO* on pCTX1 vector	This study
pCTX1-*ptsO* (H15A)	pCTX1-*ptsO* with His 15 codon mutated to Ala	This study
pCTX1-*ptsO* (3XFlag)	pCTX1-*ptsO* with 3XFlag tag at the 3’ end	This study
pCTX1-*ptsO* (H15A-3XFlag)	CTX1-*ptsO* H15A with 3XFlag tag at the 3’ end	This study
pEXG2 Δ*ptsO*	Used to make in-frame *ptsO* deletion	(4)
pEXG2 Δ*ptsN*	Used to make in-frame *ptsN* deletion	(4)
pEXG2 Δ*lasR* (PA14)	Used to make in-frame *lasR* deletion with *rsaL* intact	Kostylev, unpublished
pEXG2 Δ*fruB*	Used to make in-frame *fruB* deletion	(5)
pEXG2 Δ*nagF*	Used to make in-frame *nagF* deletion	(5)

Supplemental references

1. Chugani S, Greenberg EP. LuxR homolog-independent gene regulation by acyl-homoserine lactones in *Pseudomonas aeruginosa*. Proceedings of the National Academy of Sciences of the United States of America. 2010;107(23):10673-8.
2. Rahme LG, Stevens EJ, Wolfort SF, Shao J, Tompkins RG, Ausubel FM. Common virulence factors for bacterial pathogenicity in plants and animals. Science. 1995;268(5219):1899-902.
3. Abisado RG, Kimbrough JH, McKee BM, Craddock VD, Smalley NE, Dandekar AA, et al. Tobramycin adaptation enhances policing of social cheaters in *Pseudomonas aeruginosa*. Applied and environmental microbiology. 2021;87(12):e0002921.
4. Cabeen MT, Leiman SA, Losick R. Colony-morphology screening uncovers a role for the *Pseudomonas aeruginosa* nitrogen-related phosphotransferase system in biofilm formation. Mol Microbiol. 2016;99(3):557-70.
5. Underhill SAM, Pan S, Erdmann M, Cabeen MT. PtsN in *Pseudomonas aeruginosa* is phosphorylated by redundant upstream proteins and impacts virulence-related genes. J Bacteriol. 2023;205(5):e00453-22.
6. Simon R, Priefer U, Pühler A. A Broad Host Range Mobilization system for in vivo genetic engineering: transposon mutagenesis in Gram negative bacteria. Bio/Technology. 1983;1(9):784-91.
7. Feltner JB, Wolter DJ, Pope CE, Groleau MC, Smalley NE, Greenberg EP, et al. LasR variant cystic fibrosis isolates reveal an adaptable quorum-sensing hierarchy in *Pseudomonas aeruginosa*. mBio. 2016;7(5).
8. Miller WG, Leveau JH, Lindow SE. Improved *gfp* and *inaZ* broad-host-range promoter-probe vectors. *Mol Plant Microbe Interact*. 2000;13(11):1243-50.
9. Hoang TT, Kutchma AJ, Becher A, Schweizer HP. Integration-proficient plasmids for *Pseudomonas aeruginosa*: site-specific integration and use for engineering of reporter and expression strains. *Plasmid*. 2000;43(1):59-72.
10. Meisner J, Goldberg JB. The Escherichia coli *rhaSR-PrhaBAD* inducible promoter system allows tightly controlled gene expression over a wide range in *Pseudomonas aeruginosa.* Applied and Environmental Microbiology. 2016;82(22):6715-27.
11. Rietsch A, Vallet-Gely I, Dove SL, Mekalanos JJ. ExsE, a secreted regulator of type III secretion genes in *Pseudomonas aeruginosa*. Proceedings of the National Academy of Sciences of the United States of America. 2005;102(22):8006-11.
12. Wellington S, Greenberg EP. Quorum sensing signal selectivity and the potential for interspecies cross talk. mBio. 2019;10(2):10.1128/mbio.00146-19.
13. Cruz RL, Asfahl KL, Bossche SVd, Coenye T, Crabbé A, Dandekar AA. RhlR-regulated acyl-homoserine lactone quorum sensing in a cystic fibrosis isolate of *Pseudomonas aeruginosa.* mBio. 2020;11(2):10.1128/mbio.00532-20.
14. Scholz RL, Greenberg EP. Positive Autoregulation of an acyl-homoserine lactone quorum-sensing circuit synchronizes the population response. mBio. 2017;8(4):e01079-17.
